# Effects of Bonding Treatment and Ball Milling on W-20 wt.% Cu Composite Powder for Injection Molding

**DOI:** 10.3390/ma14081897

**Published:** 2021-04-10

**Authors:** Mingliang Ouyang, Cuiping Wang, Huayu Zhang, Xingjun Liu

**Affiliations:** 1 School of Materials Science and Engineering, Harbin Institute of Technology, Shenzhen 518055, China; 16b954081@stu.hit.edu.cn (M.O.); wangcp@xmu.edu.cn (C.W.); 2 Institute of Materials Genome & Big Data, Harbin Institute of Technology, Shenzhen 518055, China; 3 School of Materials Science and Engineering, Xiamen University of Technology, Xiamen 361024, China

**Keywords:** tungsten–copper composite, bonding treatment, ball milling, powder injection molding

## Abstract

W-20 wt.% Cu pseudo-alloys were produced via powder injection molding (PIM) with powders prepared thermochemically. Bonding treatment and ball milling (BTBM) were used, and the effects of BTBM on the characteristics of the powders, rheological properties of the feedstock, shrinkage and properties of the sintered samples were studied. The morphology of the powder changed from extremely agglomerated small particles to pebble-shaped smooth large particles which were composed of several small particles combined tightly. The tap density increased from 3.25 g/cm^3^ to 7.22 g/cm^3^, and the specific surface area decreased from 0.86 m^2^/g to 0.45 m^2^/g. The critical powder loading of the feedstock increased from 45 vol.% to 56 vol.% due to the change in powder characteristics, thereby improving densification and dimension precision. For the PIM samples sintered at 1290 °C for 120 min in a hydrogen gas, the oversizing factor decreased from 1.297 to 1.216, and the dimension fluctuation ratio decreased from ±0.61% to ±0.33%. At the same time, the relative density increased from 97.8% to 98.6%, the thermal conductivity increased from 218 W/(m·K) to 233 W/(m·K), and the average coefficients of thermal expansion were roughly similar, within the range of 8.43–8.52 × 10^−6^/K.

## 1. Introduction

Tungsten–copper (W-Cu) pseudo-alloys are widely used in the manufacturing of electrical contact materials, electrodes, thermal management devices, and conductive inks for ceramic metallization due to their high electrical conductivity, high thermal conductivity, low coefficient of thermal expansion (CTE), and many other excellent properties [1,2,3,4,5,6,7,8,9,10]. With the rapid development of microelectronics and packaging technology, W-Cu heat sink components become smaller, and their shapes become more complex. In theory, the thermal conductivity of W-Cu pseudo-alloys is very sensitive to density, which is extremely important for their application as heat sink materials in the electronics industry. However, many problems, such as difficulties in achieving high densities, copper bleed-outs, coarse and uneven microstructure, are found in the production of these dense W-Cu components. Hence, economically producing W-Cu parts with high densities and complex shapes using traditional methods remains challenging [11,12,13]. Powder injection molding (PIM) is an efficient method used in mass production of small and complicated parts. Metal parts with high melting point, such as tungsten and molybdenum, can be produced efficiently by using PIM technology [14,15,16,17,18,19,20,21]. Powder properties, such as micromorphology, specific surface area, particle size and distribution, free slope angle, and tap density, have considerable effects on the powder loading and rheological behavior of the PIM feedstock, thereby affecting the shrinkage and dimensional stability during sintering. The agglomeration of particles degrades the rheological properties of the feedstock resulting in filling defects in the injection molding process [22,23,24,25].

Ultrafine W-20% Cu powders prepared thermochemically [26,27] have many advantages, such as good sinterability, high purity, excellent properties after sintering, and negligible copper bleed-outs during sintering. These features are attractive for PIM to manufacture delicate W-Cu parts. However, ultrafine powders agglomerate easily, and the feedstock made by ultrafine W-Cu powder has poor fluidity and low powder loading, thereby leading to the instability of product dimensions and performance. Many methods are reported to improve the PIM of agglomerated powder. Ouyang et al. [15] investigated the effect of jet milling on W-10 wt.% Cu powders for PIM. The results show that jet milling disperses the agglomerated ultrafine powder effectively, and the treated powder has a high tap density, thereby increasing the powder loading of feedstock and stabilizes the dimensions of sintered products. Fan et al. [28] used ball milling and stearic acid to remove agglomeration from fine powders, effectively improving the compatibility and powder loading of feedstock. Ma et al. [29] applied ball milling to mix carbonyl iron and carbonyl nickel powders. Homogeneous and dispersion powders with high tap densities are obtained, thereby increasing the powder loading of feedstock and dimensional accuracy of sintered samples.

In this work, bonding treatment and ball milling (BTBM) were used to improve the powder characteristics of ultrafine W-20% Cu powders. The effects of BTBM on the powder characteristics, rheological behavior of feedstock, oversizing factor (OSF), dimensions fluctuations and properties of the sintered samples were investigated.

## 2. Materials and Methods

In this work, ammonium paratungstate (APT, (NH_4_)_10_(H_2_W_12_O_42_)·4H_2_O), copper nitrate (Cu(NO_3_)_2_·3H_2_O), citric acid (C_6_H_8_O_7_·2H_2_O), and stearic acid (CH_3_(CH_2_)_16_COOH) were obtained from Sinopharm Chemical Reagent Co., Ltd. (Shanghai, China). All chemicals used were of analytical reagent grade. W-20% Cu ultrafine powders were synthesized through dissolution, spray drying, calcination, and subsequent hydrogen reduction. APT (11.36 kg), Cu(NO_3_)_2_·3H_2_O (7.61 kg), and appropriate amounts of citric acid were added into 60 L of deionized water. The solution was spray dried and then calcined at 600 °C for 4 h to obtain the oxide mixture after stirring at 90 °C for 30 min. W-20% Cu ultrafine powders were obtained through the following hydrogen reduction at 800 °C for 3 h in a flowing hydrogen atmosphere. Bonding treatment was conducted in a tube furnace under a flowing hydrogen atmosphere. The temperature was raised to 1200 °C at a rate of 3 °C/min for 30 min and then cooled to room temperature. The bonding treatment powders were then ball milled under the protection of argon for 12 h with a rod to powder ratio of 5:1 and rotation speed of 60 rpm. A polyurethane pot and tungsten rod of Φ7 mm × 5 mm were used to prevent impurities during ball milling. In this study, the feedstock was prepared by mixing a wax-based binder and W-20% Cu powders. The binder is composed of 60 wt.% paraffin wax, 17 wt.% polypropylene, 20 wt.% high-density polyethylene, and 3 wt.% stearic acid. It ensures high flowability of the feedstock and determines the stability of the molding process in mass production [30,31]. The feedstock was mixed with a torque rheometer at 150 °C and then injected into the green parts with different molds. In the next step, the binder was removed through extraction and subsequent thermal decomposition. Extraction was carried out in heptane at 50 °C for 3 h, thermal decomposition was carried out in a tube furnace under the protection of flowing hydrogen, the tube furnace was heated to 800 °C at the rate of 1 °C per minute and kept for 2 h. After thermal decomposition, the parts were sintered at 1290 °C for 120 min under the protection of flowing hydrogen.

The free slope angle and tap density of powders were tested using a powder comprehensive characteristic tester (BT-1000, Dandong, China). The particle size distribution was tested using a laser scattering measurement device (Malvern Mastersizer 2000, Cambridge, UK). The specific surface area was measured using a surface area and porosity analyzer (TriStar II, Micromeritics, Norcross, GA, USA). The mixing torque of the feedstocks was measured using a torque rheometer (Changkai CTR-100, Shanghai, China). The density was calculated on the basis of Archimedes’ principle. The chemical compositions were characterized via an inductively coupled plasma emission spectrometer (ICP-OES, ICAP 7400, Waltham, MA, USA). The carbon content was determined using a carbon and sulfur analyzer (LECO CS-200, Stevensville, MI, USA). The oxygen content was determined using a combined oxygen and nitrogen analyzer (Horiba EMGA-820, Tokyo, Japan). The microstructure of powders and sintered composites were analyzed using a scanning electron microscope (SEM, ZEISS EVO18, Oberkochen, Germany) at an operating voltage of 20.0 kV. The thermal conductivity was tested using a laser flash apparatus (LFA457, Netzsch, Johannesburg, Germany) in argon at room temperature. The average CTE value was measured using a thermal mechanical analyzer (TMA402F3, Netzsch, Germany) in argon, and the temperature varied from 25 °C to 300 °C at a heating rate of 5 °C/min.

## 3. Results and Discussion

**Figure 1:**
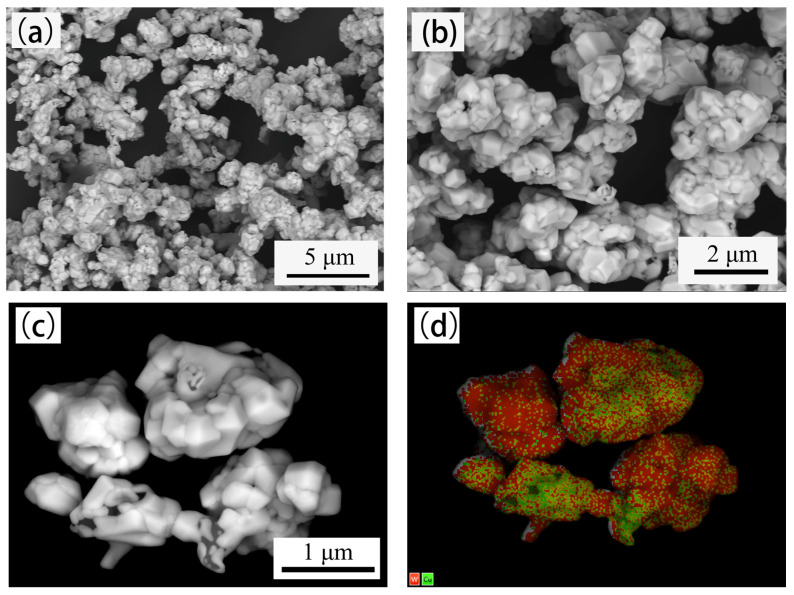
Micromorphology of W–20% Cu powders synthesized by thermochemical method (powder A). (**a**) 5000× magnification; (**b**) 10,000× magnification; (**c**) 25,000× magnification; (**d**) EDX mapping analysis of (**c**).

Figure 1 shows the micromorphology of W-20% Cu powders synthesized thermochemically (Powder A). The powders exhibit a uniform polyhedral structure with average particle size of around 0.8–1.0 μm. Strong agglomeration was observed, as shown in Figure 1a–c. At high magnification, the powders clearly have the structure of tungsten-coated copper, which is identified through energy-dispersive X-ray mapping analysis (Figure 1d). The chemical composition of Powder A obtained by ICP-OES analysis is presented in Table 1, and its Cu concentration is 20.4613 wt.%, which is close to the design value (i.e., 20 wt.%).

**Figure 2:**
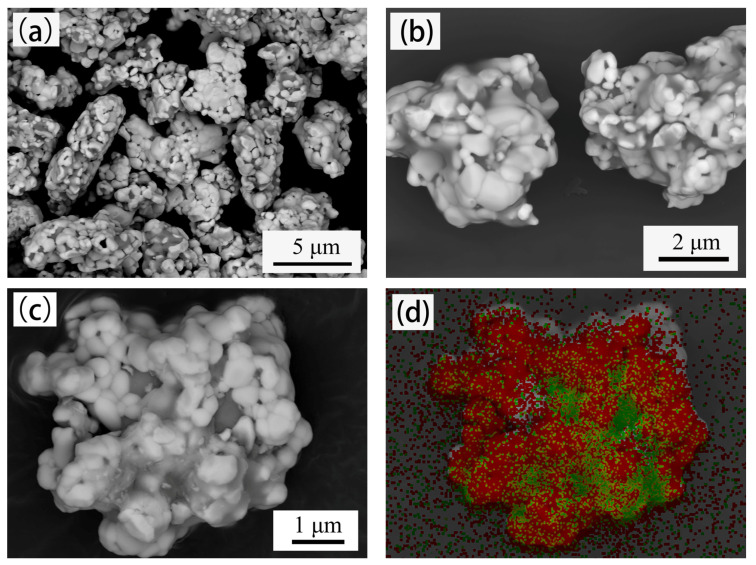
Micromorphology of W–20% Cu powders after bonding treatment and ball milling (BTBM, Powder B). (**a**) 5000× magnification; (**b**) 10,000× magnification; (**c**) 15,000× magnification; (**d**) EDX mapping analysis of (**c**).

Figure 2 shows the micromorphology of W–20% Cu powders after BTBM (Powder B). The microstructure of the powders changed greatly, Powder B is pebble shaped, and its surface is smooth, as displayed in Figure 2a,b. The average particle size of Powder B is approximately 3–10 μm, and each particle is composed of several small ultrafine particles with the structure of tungsten-coated copper, as shown in Figure 2c,d. The ultrafine powders are tightly combined in large pebble-shaped particles without gaps or voids between them. Inside the pebble-shaped particles of Powder B, sintered necks are formed between ultrafine-particles, and part of the copper penetrates between the small particles, as shown in Figure 2c. The bonding of sintered necks and copper penetration caused the ultrafine particles to clump together. During the subsequent ball milling, part of the bonding is destroyed again. However, some strong joints are not destroyed. Thus, these combined ultrafine particles form a new large particle. During ball milling, as the large new particles are constantly impacted by the tungsten rod, the gaps and voids between the ultrafine particles gradually disappear, and the outer surface gradually becomes rounded and pebble-shaped, as shown in Figure 2. The chemical composition of Powder B obtained by ICP-OES analysis is presented in Table 1. The Cu concentration is 20.4037 wt.%, which is equal to Powder A. The content of carbon and oxygen increased as expected due to the wearing of the milling pot, but they were eliminated after sintering in hydrogen, as shown in Table 1.

**Figure 3:**
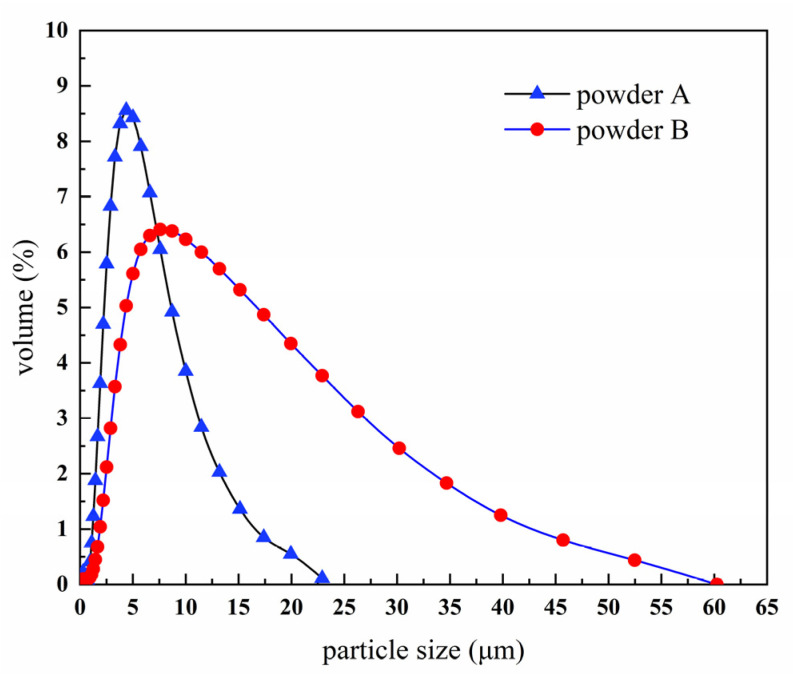
Particle size distribution of W-20% Cu powders.

The results of powder size distribution by the laser particle size analyzer are shown in Figure 3. The average particle size increased, and a wide particle size distribution is obtained after BTBM. As shown in Table 2, the values of D_10_, D_50_, and D_90_ increase from 2.143, 4.512, and 8.669 μm to 2.863, 8.011, and 22.830 μm. Particle size distribution is an important technological parameter for PIM, the viscosity of feedstock is affected by different particle size distributions, and the particle size distribution slope parameter called S_w_ can be calculated by using Equation (1).

(1) $$S_{w}=2.56/\log\left(D_{90}/D_{10}\right)$$

The parameter S_w_ is the slope of the log-normal cumulative distribution and it is similar to a coefficient of variation or standard deviation. The higher the value of S_w_, the narrower particle distribution is. Narrow particle size distribution (S_w_ of 4–7) of powder will generally result in high viscosity of a feedstock, whereas wide particle size distribution (S_w_ of 2–4) means low viscosity of a feedstock and easier filling of a mold [32]. S_w_ of powders were calculated to predict the suitability for injection molding, the S_w_ of Powder A is 4.22, and the S_w_ of Powder B is 2.84, which indicates that the feedstock mix by Powder B has lower viscosity, so Powder B is more suitable for PIM.

With the increase of particle size, the specific surface area decreased from 0.86 m^2^/g to 0.45 m^2^/g, as shown in Table 2. In the feedstock, each powder particle should be covered by a very thin film of binder, a smaller specific surface area indicates that the powders need less binder to wet the powder particles, and the feedstock consequently will flow with the lower viscosity. At the same time, a smaller specific surface area is certainly linked to an reduce of the friction forces between the powder and binder, which will lead to the decrease of viscosity of the feedstock.

After BTBM, the tap density increased from 3.25 g/cm^3^ to 7.22 g/cm^3^, and the free slope angle decreased from 52.19° to 40.53°, as shown in Table 2. The changes occurred due to the change in powder morphology and particle size distribution, as shown in Figure 1, Figure 2 and Figure 3. The loose and agglomerated ultrafine powders became dense and large pebble-shaped particles with smooth surface, and the particle size distribution became wider. The increase in tap density indicates the decrease in powder volume, while the decrease in free slope angle indicates the decrease in friction between powders. These changes are helpful to improve the powder loading and flowability of the feedstock.

**Figure 4:**
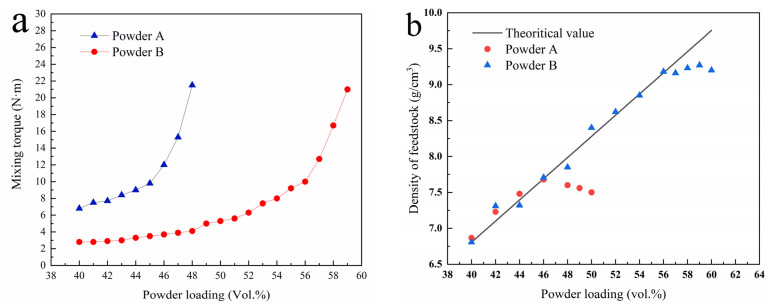
(**a**) Mixing torque of feedstock variation with the powder loading; (**b**) density of feedstock variation with the powder loading.

In theory, the powder surface of a qualified feedstock should be completely wrapped with binder, and no porosity should exist in the feedstock. The volume ratio of powder is called the powder loading and the maximum powder loading without porosity is called the critical powder loading. A high critical powder loading is always expected to obtain high dimensional accuracy. The critical powder loading is significantly affected by the powder characteristics. The mixing torque is the feedback of energy consumed by the powder dispersed in the adhesive, indicating the viscosity of the mixture. Therefore, evaluating the critical powder loading by monitoring the torque variation during the feedstock mixing is a conventional method [33,34]. The variation of mixing torque with powder loading of feedstock is shown in Figure 4. In the early stage, the mixing torque increases slowly with the increase in powder loading. When the powder loading is greater than a critical point, the mixing torque increases significantly, usually corresponding to the critical powder loading of the feedstock. As shown in Figure 4a, the critical points for powders A and B are 45 and 56 vol.%, respectively. When the powder loading is equal, the mixing torque of powder B is lower than that of powder A, indicating that less mixing energy is required for powder B to obtain a homogeneous feedstock. Compared with Powder B, Powder A has serious agglomeration, smaller particle size, larger specific surface area, and larger free slope angle, as shown in Table 2. These conditions definitely result in an increase in friction forces between the powders and the binder and lead to an increase in viscosity. The surface of Powder B is smoother than Powder A (Figure 1 and Figure 2), making it easier to be dispersed during mixing.

The theoretical density (ρ) of feedstock can be calculated in terms of powder loading and theoretical density of powder and binder by using Equation (2).

(2) $$\rho =\rho_{b}+\varphi \left(\rho_{p}-\rho_{b}\right),$$

where $\rho_{b}$ is the theoretical density of binder, $\rho_{p}$ is the theoretical density of powder, and φ is the powder loading.

When the powder loading is larger than the critical value, pores are found in the feedstock, and the density of feedstock is lower than the theoretical value. Figure 4b shows the feedstock density variation with the powder loading. For Powder A, the discrepancy between the measured value and the theoretical value is 45 vol.%, indicating the critical powder loading of Powder A is 45 vol.%. After BTBM, the critical powder loading of Powder B is 56 vol.%, which is 11 vol.% higher than that of Powder A, and the result is consistent with Figure 4a.

**Figure 5:**
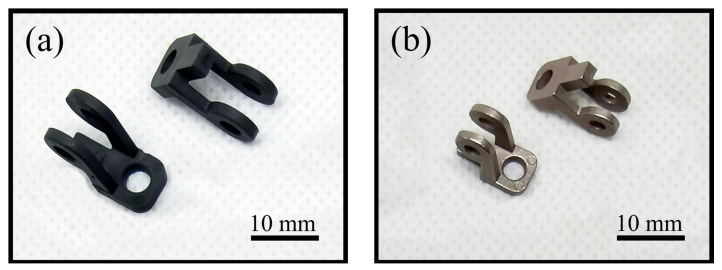
Supporter parts produced via powder injection molding (PIM) (**a**) green part; (**b**) sintered part.

**Figure 6:**
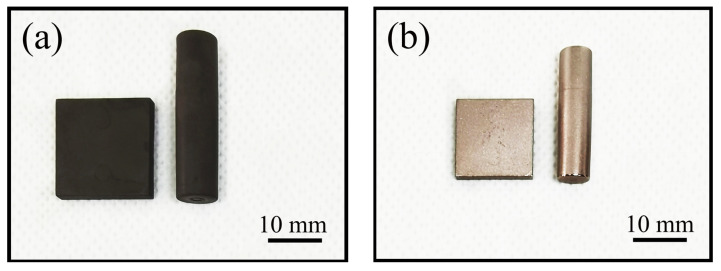
Test samples produced via PIM (**a**) green part; (**b**) sintered part after polish.

PIM is suitable for the mass production of tungsten–copper components with complicated shapes and exquisite structures. One hundred pieces of supporter parts with two long arms and side holes were injected to illustrate the excellent shaping ability of PIM, as shown in Figure 5. A square sample and a cylindrical sample were injected to test the performance of thermal conductivity and CTE, as shown in Figure 6.

Table 3 shows the effect of powder loading on the dimension precision and OSF of the supporter parts. The lengths of the cave and supporter parts were measured to evaluate the dimension fluctuation and OSF. When the powder loading increases from 45% to 56%, the OSF decreases from 1.297 to 1.216, and the dimension fluctuation ratio decreases from ±0.61% to ±0.33%. The results show that the dimensional accuracy of the products improves with the decrease in sintering shrinkage. High powder loading of feedstock reduces sintering shrinkage and improves the dimensional stability of products. 

**Figure 7:**
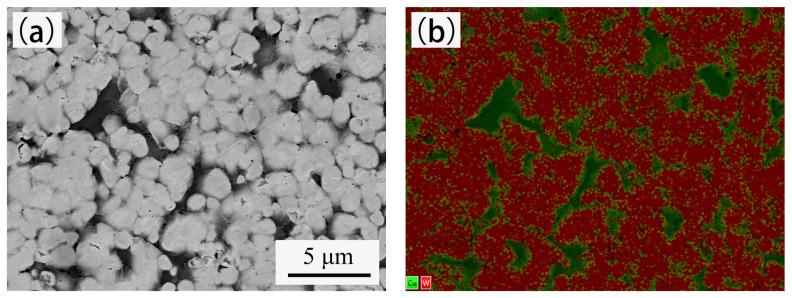

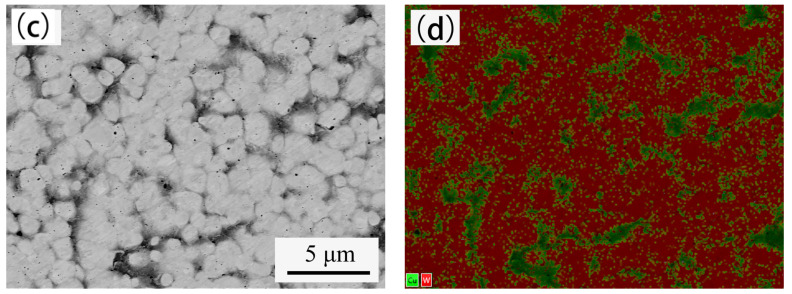
Back-scattered electron images (BEI) and elemental mapping of sintered W-20% Cu composite (**a**,**b**) via powder A; (**c**,**d**) via powder B.

Figure 7 shows the back-scattered electron images (BEI) and elemental mapping analysis of cross-section of sintered W–20% Cu composite. The red part indicates tungsten, and the green part indicates copper. The copper is evenly distributed between tungsten grains. The tungsten grains are approximately 1–2 μm, the maximum size of the copper area is less than 5 μm, and no obvious defects are detected. The densities of W–20% Cu composite sintered with powders A and B are 15.31 g/cm^3^ (97.8% of theoretical density) and 15.43 g/cm^3^ (98.6% of theoretical density), respectively, as shown in Table 4. These findings indicate that the W–20% Cu parts were well fabricated. Compared with the composite sintered by Powder A, some differences were observed in the composite sintered by Powder B, such as smaller size of single copper area, more uniform distribution of copper, and higher density, which indicate that better properties of W–20% Cu composite could be obtained after BTBM.

During heating from room temperature to the melting point of copper (1083 °C), the average grain size of initial tungsten particles determines the intensity of solid-state densification [35,36,37,38]. When the sintering temperature is higher than 1083 °C, the liquid-phase sintering begins. The tungsten particles were rearranged driven by the capillary of liquid copper, and the small tungsten particle size can accelerate the densification [36,39,40,41]. The ultrafine composite powders change into large pebble-shaped particles after BTBM due to the bonding of molten copper. However, the size of tungsten particles does not change. Thus, powders A and B exhibit similar sintering behavior. The density of W–20% Cu composite sintered by Powder B is 0.8% higher than that sintered by Powder A. This is because the powder loading of Powder B is 11 vol.% higher than that of Powder A. The shrinkage of sample from Powder A is greater than that of Powder B during sintering. The tungsten particles in the sample made from Powder B need to move a shorter distance to achieve the same density as that of Powder A. In other words, the densification of Powder B requires less energy than Powder A. Large pores are easily formed between the aggregates in Powder A, which are difficult to completely eliminate during sintering. For the above reasons, the density of W-20% Cu composite sintered from Powder B is higher than Powder A under the same sintering conditions.

The thermal conductivity and CTE are tested to study the properties of W–20% Cu composite sintered from Powders A and B. The square samples were machined into disks (Φ12.7 mm × 2.0 mm), and the cylindrical samples were machined into cylinders (Φ6.0 mm × 23.0 mm). The disk samples were used to measure the thermal conductivity, and the cylinder samples were used to measure the CTE. The test results are presented in Table 4.

Density and microstructure are the main factors affecting the properties of W-20% Cu composite. A high density and uniform microstructure usually indicate better properties [42,43,44]. As shown in Figure 7, copper is evenly distributed between tungsten particles, and tungsten particles are connected with each other to form a uniform tungsten skeleton. The microstructure determines that the W-20% Cu composite has a high thermal conductivity and a low CTE [45,46]. The thermal conductivity of sample B (233 W/(m·K)) is higher than sample A (218 W/(m·K), which is due to the higher density of sample B. The average CTE value of the sample sintered from Powder B is 8.43 × 10^−6^/K, which is similar to that of the sample sintered from Powder A (8.52 × 10^−6^/K).

## 4. Conclusions

In this study, W-20% Cu pseudo-alloys were produced through PIM using powders synthesized thermochemically. The effects of BTBM on the characteristics of powders, rheological properties of the feedstock, shrinkage during sintering, and the dimension fluctuation and properties of the sintered samples were studied. The conclusions are summarized as follows:

(1) The characteristics of ultrafine W-20% Cu powders changed greatly after BTBM, which significantly increased the powder loading, improved the fluidity of the feedstock, and then improved the properties and dimension accuracy of the PIM products.
(2) The morphology of the powder changed from extremely agglomerated small particles to pebble-shaped smooth larger particles under the combined action of bonding, impact and rolling during BTBM, and each larger particle is composed of several small ultrafine particles. The powders after BTBM have characteristics of larger particle size, wider particle size distribution, smaller specific surface area, higher tap density and smooth surface, which are beneficial to decrease the viscosity and increase the powder loading of the feedstock.
(3) The densification rate of W-20% Cu composites is mainly determined by the size of initial tungsten particles. BTBM did not increase the size of tungsten particles in W-20% Cu ultrafine powder, but eliminated the gaps and voids of ultrafine powder agglomerates, increased the powder loading of feedstock, and reduced the migration distance of tungsten particles in the sintering process. Therefore, the sintered material obtained more uniform microstructure, higher density, and better properties.

## Figures and Tables

**Table 1:** Chemical composition of W–20% Cu powders and composite.

Element	Powder A	Powder B	Sintered Composite (Powder A)	Sintered Composite (Powder B)
W/(wt.%)	Bal.	Bal.	Bal.	Bal.
Cu/(wt.%)	20.4613	20.4037	20.1537	20.1096
Cr/(wt.%)	0.0073	0.0069	0.0068	0.0057
Fe/(wt.%)	0.0041	0.0058	0.0036	0.0049
Ni/(wt.%)	0.0032	0.0028	0.0039	0.0042
Mo/(wt.%)	0.0086	0.0079	0.0067	0.0075
C/(wt.%)	0.0018	0.0373	0.0031	0.0025
O/(wt.%)	0.2650	0.3821	0.0103	0.0079

**Table 2:** Powder characteristics of W–20% Cu powders.

Powder	Particle Size Distribution			Specific Surface Area (m^2^/g)	Free Slope Angle (°)	Tap Density (g·cm^−3^)
	D_10_ (μm)	D_50_ (μm)	D_90_ (μm)			
Powder A	2.143	4.512	8.669	0.86	52.19	3.25
Powder B	2.863	8.011	22.830	0.45	40.53	7.22

**Table 3:** Effect of powder loading on dimension precision and oversizing factor (OSF) of the supporter parts.

Powder	Powder Loading (vol.%)	Cave Length (mm)	Average Length of Products (mm)	Dimension Fluctuation (mm)	Fluctuation Ratio (%)	Oversizing Factor
Powder A	45	14.80	11.41	±0.07	±0.61	1.297
Powder B	56	14.80	12.17	±0.04	±0.33	1.216

**Table 4:** Properties of the W–20% Cu composite sintered from powders A and B.

Powder	Density (g·cm^−3^)	Relative Density (%)	Coefficient of Thermal Expansion (10^−^^6^/K)	Thermal Conductivity (W/mK)
Powder A	15.31	97.8%	8.52	218
Powder B	15.43	98.6%	8.43	233

## Data Availability

The data presented in this study are available on request from the corresponding author.
